# Editorial

**DOI:** 10.1093/nc/niv001

**Published:** 2015-03-03

**Authors:** Anil K. Seth, Biyu J. He, Jakob Hohwy

**Affiliations:** Sackler Centre for Consciousness Science, School of Engineering and Informatics, University of Sussex; National Institutes of Health, National Institutes of Neurological Disorders and Stroke, National Institutes of Health.; Cognition & Philosophy Lab, Monash University.

‘It is remarkable that most of the work in both cognitive science and the neurosciences makes no reference to consciousness (or “awareness”).’ So wrote Francis Crick and Christof Koch, a quarter of a century ago, in a paper which with hindsight marked the rebirth of consciousness science as a serious enterprise (Crick and Koch, 1990). Times have changed and consciousness science has since flourished. It is now a rich multi-disciplinary enterprise engaging neuroscientists, psychologists, philosophers, computer scientists, clinicians and physicists, collaborating in the systematic investigation of the biological and physical mechanisms underlying conscious experience. Consciousness is studied in psychiatric and neurological patients, in non-human animals and in healthy human subjects, with experiments deploying increasingly powerful methodologies for acquiring, analysing and connecting first-person behavioural, neurophysiological and genetic data. The launch of *Neuroscience of Consciousness* reflects the maturity of this rigorous and empirically grounded approach to the science of subjective experience. As its Editors we share the conviction that natural science has much to say about consciousness, and we look forward to this story unfolding within this new *Journal*.

People have been wondering about consciousness since they have wondered about anything. Hippocrates long ago identified the brain as the primary organ of experience (though Aristotle did not). Much later, Descartes codified what we now recognize as the ‘hard problem’ of relating matter and consciousness (Chalmers, 1996). At the birth of scientific psychology, midwived by William James and Wilhelm Wundt at the close of the 19th century, consciousness was the central explanatory target (James, 1892). But through the 20th century the influence of behaviourism shifted the goal of psychology to the prediction and control of behaviour, with the study of consciousness pushed to the sidelines. The suppression of consciousness continued even through the rise of cognitive science from the 1960s, albeit with important exceptions here and there. The situation changed dramatically with the rise of functional brain imaging in the 1990s, allowing researchers to examine the living brain while their subjects performed different tasks and had different experiences. At the same time, authorities in biology like Crick and Gerald Edelman (having already won their Nobel prizes) turned their attention explicitly to consciousness (Edelman, 1989; Crick and Koch, 1990), motivated in part by early examples such as the ‘split brain’ studies of Roger Sperry and Mike Gazzaniga (Gazzaniga *et al.,* 1962). Theoretical models like Bernard Baars’ global workspace theory (Baars, 1988), coupled with advances in psychophysical and neurophysiological methods, enabled researchers to look for ‘neural correlates’ of consciousness as a first step towards its naturalization (Crick and Koch, 1990; Metzinger, 2000). Concepts of ‘neuronal synchrony’ provided the first plausible explanations linking the dynamics of brain activity to properties of conscious experience (Gray *et al.,* 1989). In parallel with these developments, there have been increasing efforts to understand the neurobiology of psychopathology in terms of consciousness (Frith, 1979). There has since been a flowering of research aimed directly at understanding the biological basis of consciousness, which has been well covered in several recent reviews (Tononi and Koch, 2008; Dehaene and Changeux, 2011; van Gaal and Lamme, 2012; Boly *et al.,* 2013; Brugger and Lenggenhager, 2014).

A challenge facing consciousness science is the lack of a consensual definition for consciousness. This situation is, however, not unusual in science, where definitions, theories and experiments often evolve in parallel rather than in a nice orderly sequence. But perhaps because we all have conscious experiences ourselves, intuitions about what consciousness ‘is’ are commonplace and often strongly held. In one sense these intuitions can be summarized very simply, by saying that for a conscious organism ‘there is something it is like to be that organism’ (Nagel, 1974). Or one can simply indicate that consciousness (for humans) is what disappears when we fall into a dreamless sleep and what returns the next morning when we wake up. For conscious organisms there exists a continuous (though interruptible) stream of conscious scenes or experiences—a phenomenal world—which has the character of being subjective and private.

Beyond these basic statements, opinions differ about how to characterize consciousness as an explanatory target. One increasingly accepted distinction is between conscious ‘level’ (how conscious the organism is) and conscious ‘content’ (being conscious of this rather than that). Another is between consciousness of the ‘world’ and of the ‘self’, which is sometimes related to a distinction between ‘primary’ and ‘higher-order’ consciousness (Edelman, 2003). We do not wish to impose any particular way of thinking here, beyond suggesting that our common sense or folk intuitions about what consciousness ‘is’ should be open to revision, as theories and experiments develop. At the same time, a successful science of consciousness must distinguish between rigorous, testable scientific ideas and those that involve more outlandish speculations (Block *et al.,* 2014). It may be helpful to remember that consciousness science, at least for now, does not need to explain why consciousness exists, to go about unravelling the biological and physical properties that underlie its many properties, in much the same way that physicists have laid bare many mysteries of the universe without accounting for the brute fact that it is there (Seth, 2010).

The main strategy within consciousness science lies in connecting objective (third-person) data about the brain and behaviour, with subjective (first-person) data about the properties of conscious experiences (including whether they are present at all). Within this broad multidisciplinary scope there is an increasing focus on the brain as the primary biological substrate for awareness. Fortunately, the brain is increasingly accessible to consciousness researchers thanks to the rapid development and sophistication of neuroimaging and brain stimulation methods in both humans and animals, and the rich empirical and theoretical literature that already connects properties of cognition and consciousness to the structure and dynamics of the nervous system. At the same time, there is increasing sophistication within methods for obtaining and analysing first-person data (Overgaard and Sandberg, 2012), and relating this data to the brain (Lutz *et al.,* 2002; Seth *et al.,* 2008; Fleming and Dolan, 2012). The time is, therefore, right for a new journal—*Neuroscience of Consciousness*—to capitalize on the momentum within neuroscience for studying consciousness.

The overall goal of *Neuroscience of Consciousness* is ‘to support the dissemination of research findings that illuminate the biological basis of consciousness in health and in disease, in humans and in other species’. While the *Journal* will maintain an emphasis on empirical neuroscience studies, its multidisciplinary foundation encourages submission of behavioural, methodological, theoretical (including modelling) and philosophical papers that exhibit a clear relevance to the biological basis of consciousness. We also emphasize a clinical dimension: the already intolerable and ever increasing burden of neurological and psychiatric illnesses, on individuals and on society, underlines the need for new interventions based on a detailed understanding of how disrupted neural mechanisms engender disordered conscious experiences.

A great variety of more specific topics fall within this general remit. Beyond the fundamental challenge of illuminating the neurobiological basis of consciousness itself, these include interactions between conscious and unconscious processes; selfhood, embodiment, and interoceptive awareness; metacognition and higher-order consciousness; emotional awareness; intention, volition, agency and awareness of actions; individual differences in consciousness; altered states of consciousness; sleep, dreaming and anaesthesia; relations between consciousness, attention and memory; social influences on consciousness; disorders of consciousness; and consciousness in infants and non-human animals. This is by no means an exhaustive list and is provided here simply for orientation rather than as a strict set of criteria. Befitting the wide range of topics, the *Journal* welcomes a variety of different article types. While we expect the majority of submissions to be research articles, we will also publish review articles, rapid communications (which benefit from expedited review), opinion pieces and spotlight commentaries.

We are delighted to join the Oxford University Press’ (OUP) family of academic journals. As part of Oxford University, OUP retains a core mission statement to support excellence in research, scholarship and education. *Neuroscience of Consciousness* is one of OUP’s flagship launches for 2015 and the Press will be supporting the *Journal* at a variety of conferences and other events this year and in the future. The *Journal* is also from the beginning fully ‘open access’, meaning that the fruits of consciousness science, as recorded in its pages, will be available to all. We are especially delighted that OUP is waiving all publication charges for at least the first year, to enable the *Journal* to hit the ground running. We are grateful to the OUP journal staff for seeing us through this far, and we are excited to continue working with them.

We are equally excited about our official partnership with the Association for the Scientific Study of Consciousness (ASSC). The ASSC has long been the premier academic society promoting research into the nature, function and underlying mechanisms of consciousness. There is a shared commitment between the ASSC and the *Journal* on rigorous empirically testable approaches, informed by (and informing) work of a more theoretical and philosophical nature. There is also a strong overlap in our target communities, with the ASSC’s membership including researchers in cognitive science, medicine, neuroscience, philosophy and other relevant disciplines in the sciences and humanities (see www.theassc.org for more on the ASSC). It bears emphasizing that the historical balance within the ASSC between neuroscience, psychology and philosophy, is fully embedded in the multidisciplinary scope of *Neuroscience of Consciousness*, and we thank the ASSC board for their constructive engagement with the *Journal*.

Being a new journal we are still in the process of assembling an Editorial Board. Our aim is for the board to represent the best expertise in consciousness science internationally, spread across all the participating disciplines, and with as broad an international reach as possible. Already, the three primary editors—and authors of this Editorial—are each resident in a different continent, ensuring a global coverage. We are very grateful to those scholars of consciousness who have already agreed to join. Their names are listed on the *Journal* website http://nc.oxfordjournals.org/.

These are exciting times in the science of consciousness and 2015 is an auspicious year to embark on this new adventure. One hundred years ago Einstein published his equations of general relativity, sparking a transformation in our understanding of the nature and properties of the universe. Neuroscience is of course much younger than physics, the brain is an object of extraordinary complexity (with complexity science only rigorously developed in applied mathematics for less than a century), and, at first glance, the phenomenon of consciousness seems metaphysically resistive in a way that many targets of scientific analysis are not. Hence, we are still a long way off a breakthrough comparable to that of physics. Nonetheless, the neuroscience of consciousness is flourishing, and stands poised to offer deep and important insights about our ‘inner’ universe. We anticipate that the years and decades ahead will transform our understanding of the neurobiological basis of consciousness, and with it the way we see ourselves in nature. We look forward to part of this story being told within the pages of *Neuroscience of Consciousness*.
